# Risk factors for the development of subretinal fluid in the fellow eyes of patients with chronic central serous chorioretinopathy

**DOI:** 10.1111/aos.70103

**Published:** 2026-02-12

**Authors:** Helena M. A. Feenstra, Tahir Cakar, Joost Brinks, Camiel J. F. Boon, Elon H. C. van Dijk

**Affiliations:** ^1^ Department of Ophthalmology Leiden University Medical Center Leiden the Netherlands; ^2^ Department of Ophthalmology Amsterdam University Medical Center Amsterdam the Netherlands; ^3^ Department of Vitreoretinal Surgery Rotterdam Eye Hospital Rotterdam the Netherlands

**Keywords:** central serous chorioretinopathy, fellow eye, natural disease course, pachychoroid pigment epitheliopathy, retinal pigment epithelium, subretinal fluid

## Abstract

**Purpose:**

To assess the risk of subretinal fluid (SRF) development in the fellow eye of chronic central serous chorioretinopathy (cCSC) patients with unilateral SRF at baseline.

**Methods:**

Medical records of 334 presumed cCSC patients were retrospectively reviewed. Multimodal imaging was evaluated, and the available optical coherence tomography (OCT) scans were screened for SRF development in the fellow eye during follow‐up.

**Results:**

Of 213 confirmed cCSC cases, 74 (35%) had bilateral SRF at baseline. Among 102 patients with unilateral SRF that met all inclusion criteria, 7/102 (7%) developed SRF in the fellow eye over a mean follow‐up of 20.4 months. Retinal pigment epithelial (RPE) alterations in the fellow eye were present at baseline in 7/7 (100%) who developed SRF, compared to 59/95 (62%) in those who did not (*p* = 0.042). Subfoveal choroidal thickness (SFCT) was higher in the SRF‐developing group (482.3 vs. 365.9 μm; *p* = 0.306), although not significantly. Hyperautofluorescence on fundus autofluorescence (FAF) at baseline occurred in 6/7 (86%) of the SRF‐developing group versus 25/91 (28%) who did not (*p* = 0.004). The ellipsoid zone was interrupted in 5/7 (71%) of SRF‐developing patients versus 28/95 (30%) who did not develop SRF (*p* = 0.005).

**Conclusion:**

The risk of SRF development in the fellow eye of cCSC patients with unilateral SRF is relatively low over a follow‐up period of almost 2 years. No statistically significant risk factors were identified after correction for multiple testing; however, certain trends were observed.

## 1 INTRODUCTION

Central serous chorioretinopathy (CSC) is a chorioretinal disease, which is characterized by the presence of subretinal fluid (SRF), usually located under the macula (Feenstra et al., 2024). This accumulation of SRF may cause visual symptoms such as vision loss, metamorphopsia, and diminished colour and contrast vision. These visual symptoms may severely impact the quality of life of CSC patients, who are often relatively young (Breukink et al., 2017). CSC may be categorized as either acute or chronic CSC (cCSC), with acute CSC usually resolving spontaneously within 3–4 months, whereas cCSC may persist and lead to irreversible vision loss (Mohabati et al., 2018; Mohabati et al., 2020; Mrejen et al., 2019). The pathophysiology of CSC is not yet clearly understood (Feenstra et al., 2024; Kaye et al., 2020). However, choroidal abnormalities have been hypothesized to be an important underlying factor, with subsequent damage to the retinal pigment epithelium (RPE) (Feenstra et al., 2024; Spaide et al., 2022). The most pronounced risk factors for CSC include age, male sex, pregnancy, and corticosteroid use (Feenstra et al., 2024). Currently, photodynamic therapy (PDT) is the treatment of choice for cCSC, as two large, randomized controlled trials have shown superiority over micropulse laser treatment and oral eplerenone treatment (PLACE and SPECTRA trials) (Feenstra, van Dijk, Rijssen, Tsonaka, Diederen, Hoyng, et al., 2022; Feenstra, van Dijk, Rijssen, Tsonaka, Diederen, Schlingemann, et al., 2022; van Dijk et al., 2018, 2022; van Rijssen et al., 2019, 2022).

Although visual symptoms in patients with cCSC usually present unilaterally, bilateral abnormalities on fluorescein angiography (FA) have been found to be present in up to 42% of patients (Bujarborua et al., 2005; Gackle et al., 1998; Levine et al., 1989). Bilateral SRF presence in CSC has been found to occur in up to 47% of patients. Importantly, these numbers vary (Bujarborua et al., 2005; Ersoz et al., 2018; Singh et al., 2023). Reported risk factors for bilateral disease activity include older age, RPE alterations on FA or fundus autofluorescence (FAF) and hyperfluorescent choroidal areas on indocyanine green angiography (ICGA) (Bousquet et al., 2021; Pauleikhoff et al., 2024; Spaide et al., 1996). Bilateral disease activity may negatively impact the visual functioning of patients even more, and it is therefore of great importance to assess the risk of SRF development in fellow eyes without the presence of SRF at baseline.

Currently, insufficient knowledge is available to adequately counsel cCSC patients about the risk of disease development in the fellow eye. Therefore, the aim of this study was to assess the risk of development of SRF in the fellow eye in unilateral CSC and to describe risk factors for the SRF development in that fellow eye.

## 2 MATERIALS AND METHODS

This retrospective cohort study was conducted at the Department of Ophthalmology of the Leiden University Medical Center in Leiden in the Netherlands. This study adhered to the tenets of the Declaration of Helsinki. Institutional Review Board/Ethics Committee approval was obtained from the Medical Ethical Committee Leiden—The Hague before the start of the study.

### 2.1 Participants

**FIGURE 1:**
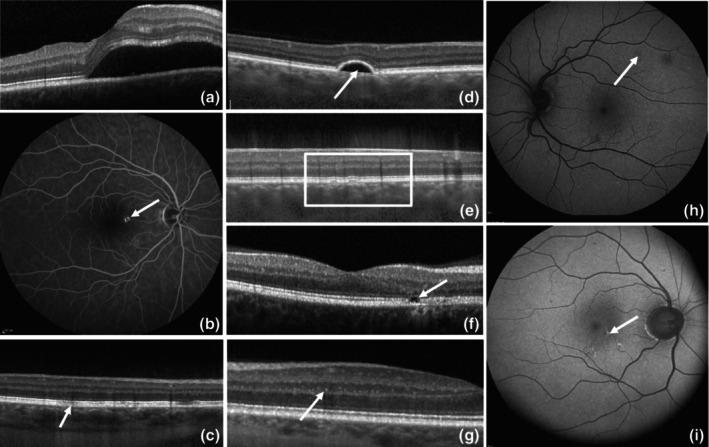
Morphological features assessed on multimodal imaging in different chronic central serous chorioretinopathy patients at baseline visit. The optical coherence tomography (OCT) scans show subretinal fluid (a), retinal pigment epithelium (RPE) attenuation (c, arrow), dome‐shaped and flat, irregular RPE detachments (d, arrow and e, respectively). On early‐phase fluorescence angiography, a window defect is visible (b, arrow). On OCT, an interrupted ellipsoid zone (f, arrow) and hyper‐reflective foci (g, arrow) are present. Hypo‐ (h, arrow and i) and hyperautofluorescence (i, arrow) are seen on fundus autofluorescence imaging.

Data and multimodal imaging of cCSC patients from clinical consultations in the Leiden University Medical Center were collected. This also included patients who participated in the PLACE trial (a previously published prospective treatment trial comparing half‐dose PDT to high‐density subthreshold micropulse laser in cCSC) and the SPECTRA trial (a previously published prospective treatment trial comparing half‐dose PDT to oral eplerenone treatment in cCSC) (Feenstra et al. 2022; van Dijk et al., 2018; van Rijssen et al., 2020, 2021, 2022). The current study enrolled patients diagnosed with cCSC, with SRF on the spectral domain (SD) 30° optical coherence tomography (OCT) scan at the first visit to the clinic, which was defined as the baseline visit. At baseline visit, OCT scans were analysed for the presence of SRF (Figure 1a), and patients were categorized as having bilateral or unilateral SRF. Patients with unilateral SRF were further assessed for eligibility. Additional inclusion criteria, according to the PLACE and SPECTRA trial criteria, included: ≥1 area of multifocal diffuse leakage or irregular RPE window defects on FA, corresponding hyperfluorescence on ICGA, and a follow‐up of at least 3 months (van Dijk et al., 2018; van Rijssen et al., 2022). In addition, the presence of complete multimodal imaging (OCT, FA and ICGA) and clinical examination at baseline visit or 1 month prior to baseline was required (Table 1). Multimodal imaging from other (referring) institutions within 1 month of baseline visit was only accepted if they met the required criteria. Exclusion criteria for the current study were myopia exceeding 6.0 diopters, presence of a macular neovascularization at baseline visit, presence of other retinal diseases that could lead to or contribute to the SRF occurrence, and lastly, insufficient quality of multimodal imaging (Table 1).

**TABLE 1:** Inclusion and exclusion criteria of chronic central serous chorioretinopathy patients who were enrolled in the current study.

Inclusion criteria	Exclusion criteria
Unilateral cCSC without SRF in the fellow eye on the OCT scan at baseline visit ≥1 area of multifocal diffuse leakage or irregular RPE window defects on FA and corresponding hyperfluorescence on ICGA Follow‐up ≥3 months for the affected eye Complete multimodal imaging present at baseline or <1 month of baseline visit (including OCT, FA, and ICGA)	Presence of SRF in the fellow eye at baseline visit on OCT scan Drusenoid or haemorrhagic RPE detachment Myopia exceeding 6.0 diopters Presence of a macular neovascularization in the affected eye at baseline Presence of other retinal diseases that could lead to or contribute to the SRF occurrence Insufficient quality of multimodal imaging

Abbreviations: cCSC, chronic central serous chorioretinopathy; FA, fluorescein angiography; ICGA, indocyanine green angiography; OCT, optical coherence tomography; RPE, retinal pigment epithelium; SRF, subretinal fluid.

### 2.2 Procedures

The baseline characteristics, such as age, gender, ethnicity, best‐corrected visual acuity (BCVA), refractive error, history of SRF in the fellow eye, and all steroid use during the 3 months prior to the start of complaints in the affected eye, were documented. Baseline characteristics of patients with bilateral and unilateral CSC at baseline visit were compared (Table 2). Multimodal imaging, which was used for this study, included OCT, FAF, FA, ICGA and fundus photographs. An OCT scan at baseline and at least 1 follow‐up OCT (after at least 3 months) was required.

**TABLE 2:** Characteristics of chronic central serous chorioretinopathy patients with bilateral and unilateral subretinal fluid at the baseline visit of this study.

	Bilateral SRF at baseline (*n* = 74)	Unilateral SRF at baseline (*n* = 102)
Age (years)	49.6 ± 11.5	47.6 ± 9.7
Male gender	67/74 (90.5%)	85/102 (83.3%)
Caucasian ethnicity	60/74 (81.1%)	88/102 (86.3%)
Endocrinological disease present	3/74 (4.1%)	5/102 (4.9%)
Cushing's syndrome	1	0
Migraine	1	2
Other	1	3
Steroid use (during or within 3 months prior to the start of complaints in the affected eye)	18/74 (24.3%)	18/102 (17.6%)

*Note*: Data are presented as either no. (%) or mean ± standard deviation.

Abbreviation: SRF, subretinal fluid.

At baseline visit, the presence of RPE alterations in the fellow eye was scored and included any of the following morphological features: RPE detachment, or other RPE irregularities on OCT (e.g., RPE attenuation and interrupted RPE), and RPE window defects on FA (Figure 1). RPE detachment was defined as any distinct separation of the RPE from the underlying Bruch's membrane (Mrejen et al., 2013). RPE window defects on FA were defined as hyperfluorescence, which arises in the choroidal phase and remains static in size and brightness (Figure 1b). Furthermore, the specific aspects of RPE detachment (dome‐shaped or flat irregular; Figure 1d,e, respectively), interruption of the ellipsoid zone (EZ) (Figure 1f), presence of hyper‐reflective intraretinal foci (Figure 1g), and subfoveal choroidal thickness (SFCT) on OCT were documented by using all scanning planes of the macular OCT scan (Feenstra et al. 2023). SFCT on OCT was defined as the distance between the outer border of the hyper‐reflective line representing the RPE and the choroidoscleral interface measured at the central foveal depression. Lastly, the presence of hypo‐ or hyperautofluorescence on FAF at baseline was documented (Figure 1h,i).

All available follow‐up visits of CSC patients with unilateral SRF at baseline visit were analysed for the presence of SRF on OCT in the fellow eye. The presence of morphological features at baseline visit was compared between the patients who developed SRF in the fellow eye during follow‐up to the patients who did not.

### 2.3 Statistical analysis

Data were collected and stored in an online Castor database (a cloud‐based clinical data management platform). Statistical analysis was performed using IBM SPSS Statistics 25 (SPSS 25, IBM, New York, U.S.A.). Snellen and Early Treatment of Diabetic Retinopathy Study BCVA were both temporarily converted to Logarithm of the Minimum Angle of Resolution for statistical analysis. The Fisher's exact test was used to analyse the nominal variables, and the *t*‐test was used for continuous variables. A *p*‐value of 0.05 was considered to be statistically significant, but after correction for multiple testing with Bonferroni correction, *p*‐values of <0.0026 were considered statistically significant.

## 3 RESULTS

**FIGURE 2:**
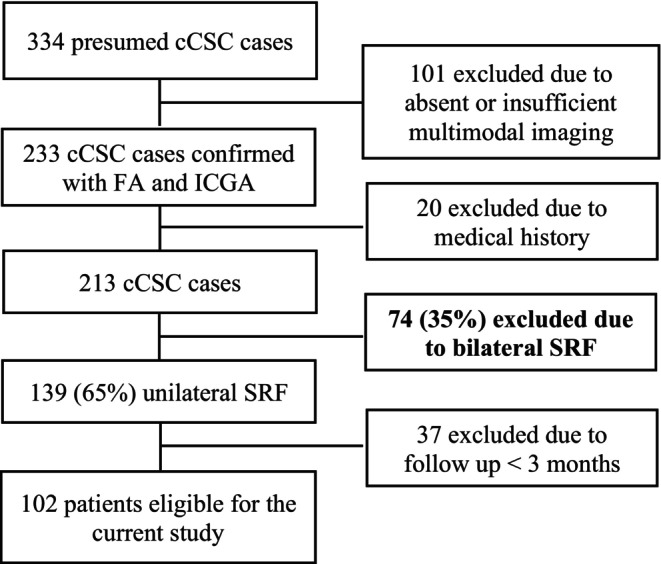
Flow chart depicting the selection criteria for the chronic central serous chorioretinopathy patients for the current study. cCSC, chronic central serous chorioretinopathy; FA, fluorescein angiography; ICGA, indocyanine green angiography; OCT, optical coherence tomography; RPE, retinal pigment epithelium; SRF, subretinal fluid.

The medical files of 334 cCSC patients who visited Leiden University Medical Center between February 2014 and October 2019 were analysed for this study (Figure 2). For the final confirmation of a diagnosis of cCSC, FA and ICGA were required, which were available in 233 patients. Twenty patients were excluded due to a medical history of other retinal diseases that could lead to or contribute to the occurrence of SRF. Out of the 213 cCSC cases confirmed on multimodal imaging, 74 (35%) had bilateral SRF on OCT at baseline. Of the patients with unilateral SRF, 37 were excluded due to insufficient follow‐up. The baseline characteristics of these included patients with unilateral SRF and the excluded patients with bilateral SRF are summarized in Table 2.

### 3.1 Development of SRF in the fellow eye in patients with unilateral SRF on OCT at baseline

**FIGURE 3:**
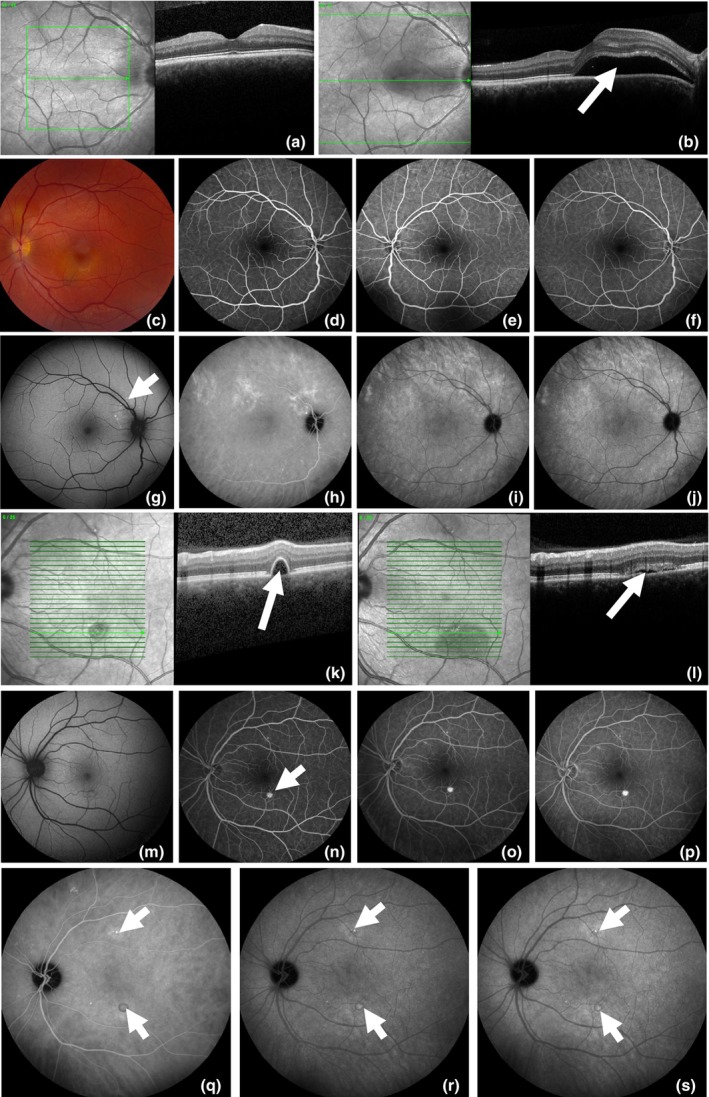
Development of subretinal fluid in the fellow eyes of two chronic central serous chorioretinopathy (cCSC) patients. Multimodal imaging of a 25‐year‐old man with cCSC (a–j). Subretinal fluid (SRF) is absent on optical coherence tomography (OCT; a) at baseline but appears beneath the fovea on OCT at 5.5 months later (b). Fundus photography (c) and early‐, mid‐, and late‐phase fluorescein angiography (d–f) taken at baseline visit do not show any abnormalities. However, hyperautofluorescence changes are visible on fundus autofluorescence (FAF; g; arrow) together with hyperfluorescence changes with an indistinct border on indocyanine green angiography (ICGA; h–j; early‐, mid‐, and late‐phase, respectively) at baseline visit, typical of diseases that are part of the pachychoroid disease spectrum. Half‐dose PDT was performed 4 months later due to persistence of SRF. Multimodal imaging of a 44‐year‐old man with cCSC (k–s). On the OCT scan, SRF is absent at baseline visit (k), but a retinal pigment epithelium (RPE) detachment outside the fovea is visible (arrow). At the location of the RPE detachment, SRF had developed at 1 year and 3 months after baseline visit (arrow, l). FAF at baseline visit does not show any pronounced abnormalities (m). However, early‐, mid‐, and late‐phase FA shows staining at the location of the RPE detachment (n, o, p, respectively). Hyper‐ and hypofluorescence changes are visible on early‐, mid‐ and late‐phase ICGA (arrows, q–r) at baseline visit, also at the location of the RPE detachment seen on OCT, and were assumed to be characteristic of diseases that are part of the pachychoroid disease spectrum. The patient was lost to follow‐up and, therefore, did not receive any treatment in his fellow eye.

Out of the patients with unilateral SRF at baseline, 102 met the inclusion criteria for this study, and multimodal imaging and clinical characteristics were analysed in detail (Figure 2). Of these cCSC patients with unilateral SRF, 85/102 (83%) were male, with a mean age of 47.6 years. The mean follow‐up time was 20.4 months. During follow‐up, 7/102 (7%) patients developed SRF in the fellow eye, after a mean time of 17.1 months after baseline visit (median, 7.1 months, range, 2.3–50 months) (Figure 3). The SRF was located at the fovea in 3/7 patients. Of the patients with subfoveal SRF, 2/3 had visual complaints (flashing lights and metamorphopsia). Out of the 4 patients with extrafoveal SRF, only 1 noticed a slight visual decline.

There were no statistically significant differences in age, gender, ethnicity, BCVA, spherical refraction, or history of previous SRF in the fellow eye when comparing cCSC patients who did and did not develop SRF in the fellow eye during the course of this study (Table 3). Steroid use within 3 months prior to the start of the first visual complaints in the affected eye was present in 4/7 (57%) in patients who developed SRF in the fellow eye during follow‐up, compared to 14/95 (15%) in patients who did not, but this difference was not significant after correction for multiple testing (*p* = 0.017).

**TABLE 3:** Baseline characteristics of chronic central serous chorioretinopathy patients included in the current study.

	Total (*n* = 102)	SRF in fellow eye during follow‐up (*n* = 7)	No SRF in fellow eye during follow‐up (*n* = 95)	*p*‐value
Age (years)	47.6 ± 9.7 (*n* = 102)	44.7 ± 10.2 (*n* = 7)	47.8 ± 9.7 (*n* = 95)	0.761^#^
Male gender	85/102 (83.3%)	7/7 (100%)	78/95 (82.1%)	0.597*
Caucasian ethnicity	88/102 (86.3%)	7/7 (100%)	81/95 (85.3%)	0.619*
Best‐corrected visual acuity in affected eye (decimal)	0.80 ± 0.66 (*n* = 99)	0.90 ± 0.72 (*n* = 7)	0.80 ± 0.66 (*n* = 92)	0.444^#^
Best‐corrected visual acuity in the fellow eye (decimal)	1.25 ± 0.75 (*n* = 99)	1.38 ± 0.10 (*n* = 7)	0.79 ± 0.12 (*n* = 92)	0.386^#^
Spherical refraction in the affected eye	+0.63 ± 1.66 (*n* = 99)	+0.43 ± 1.25 (*n* = 7)	+0.65 ± 1.69 (*n* = 92)	0.896^#^
Spherical refraction in the fellow eye	+0.31 ± 1.76 (*n* = 99)	+0.11 ± 1.20 (*n* = 7)	+0.32 ± 1.80 (*n* = 90)	0.778^#^
Previous SRF in the medical history of the fellow eye	6/102 (5.9%)	0/7 (0%)	6/95 (6.3%)	0.645*
Steroid use (during or within 3 months prior to the start of complaints in the affected eye)	18/102 (17.6%)	4/7 (57.1%)	14/95 (14.7%)	0.017*

*Note*: After correction for multiple testing with Bonferroni correction, *p*‐values <0.0026 were considered to be statistically significant. Data are presented as either no. (%) or mean ± standard deviation.

Abbreviation: SRF, subretinal fluid.

* Fisher's exact test.

^#^ Mann–Whitney *U* test.

RPE alterations at baseline were present in 7/7 (100%) of patients who developed SRF in the fellow eye versus in 59/95 (62%) of patients who did not develop SRF in the fellow eye during follow‐up (*p* = 0.042), which was not significant after correction for multiple testing (Table 4). Interruptions in the EZ on OCT at baseline were present in 5/7 (71%) patients who developed SRF in the fellow eye compared to 28/95 (30%) patients who did not develop SRF (*p* = 0.005), which was not a statistically significant difference.

**TABLE 4:** Morphological features of chronic central serous chorioretinopathy patients with unilateral subretinal fluid on multimodal imaging at baseline visit.

	Total group (*n* = 102)	Patients with SRF on OCT in the fellow eye during follow‐up (*n* = 7)	Patients without SRF on OCT in the fellow eye during follow‐up (*n* = 95)	*p*‐value
*OCT—fellow eye*				
RPE alterations^a^	66/102 (64.7%)	7/7 (100%)	59/95 (62.1%)	0.042*
RPE detachment presence	35/102 (34.3%)	4/7 (57.1%)	31/95 (32.6%)	0.228*
RPE detachment form				
Dome‐shaped (Figure 1d)	12/102 (11.8%)	1/7 (14.3%)	11/95 (11.6%)	0.595*
Flat, irregular (Figure 1e)	29/102 (28.4%)	3/7 (42.9%)	26/95 (27.4%)	0.314*
Interrupted EZ (Figure 1f)	33/102 (32.4%)	5/7 (71.4%)	28/95 (29.5%)	0.005*
Hyper‐reflective intraretinal foci (Figure 1g)	24/102 (23.5%)	1/7 (14.3%)	23/95 (24.2%)	0.477*
Subfoveal choroidal thickness (μm)	372.5 ± 127.2 (*n* = 70)	482.3 ± 186.7 (*n* = 4)	365.9 ± 121.6 (*n* = 66)	0.306^#^
*FAF—fellow eye*				
Hypoautofluorescence (Figure 1h)	48/98 (49.0%)	3/7 (42.9%)	45/91 (49.5%)	0.523*
Hyperautofluorescence (Figure 1i)	31/98 (48.9%)	6/7 (85.7%)	25/91 (27.5%)	0.004*
*FA—fellow eye*				
RPE window defects (Figure 1b)	16/95 (16.8%)	3/6 (50.0%)	13/89 (14.6%)	0.058*

*Note*: After correction for multiple testing with Bonferroni correction, *p*‐values <0.0026 were considered to be statistically significant. Data are presented as either no. (%) or mean ± standard deviation.

Abbreviations: EZ, ellipsoid zone; FA, fluorescein angiography; FAF, fundus autofluorescence; ICGA, indocyanine green angiography; OCT, optical coherence tomography; RPE, retinal pigment epithelium; SRF, subretinal fluid.

^a^ RPE alterations on OCT also include RPE detachments and RPE attenuation.

* Fisher's exact test.

^#^ Mann–Whitney *U* test.

Hyperautofluorescence on FAF at baseline occurred in 6/7 (86%) of patients who developed SRF in the fellow eye compared to 25/91 (28%) patients who did not develop SRF in the fellow eye (*p* = 0.004). There were no significant differences in hypoautofluorescence on FAF between the SRF‐developing group and the patients who did not develop SRF (3/7 (43%) vs. 45/91 (50%), *p* = 0.523). The SFCT was higher at baseline in the group of patients who developed SRF compared to the patients who did not (482.3 μm; *n* = 4 vs. 365.9 μm; *n* = 66, respectively, *p* = 0.306); however, this finding was not statistically significant.

At baseline visit, an RPE detachment was recorded in 35/102 (34%) patients, in 4/7 (57%) patients who developed SRF, compared to 31/95 (33%) patients who did not develop SRF (*p* = 0.228). There were no significant differences in the type of RPE detachments (dome‐shaped or flat‐irregular) or hyper‐reflective intraretinal foci on OCT of the fellow eye, comparing both patient groups (Table 4).

## 4 DISCUSSION

Although typical cCSC abnormalities on multimodal imaging are often seen bilaterally, most cases do not develop SRF in the fellow eye when unilateral SRF is observed at presentation. Thus far, there has been a lack of knowledge on the risk of development of SRF in the fellow eye of cCSC patients with unilateral SRF.

In the current study, we found that SRF in both eyes is present at baseline in 35% of cCSC patients. This is in line with the available literature, with numbers varying from 35% to 47% (Ersoz et al., 2018; Singh et al., 2023). Out of the 102 included cCSC patients with unilateral SRF at baseline, only 7/102 (7%) developed SRF during follow‐up after a mean time of 17.1 months after baseline. This finding suggests that the risk of SRF development in the fellow eye is relatively low when there is no SRF at baseline. Shinojima et al. reported the development of SRF in the fellow eye in 19% of undefined CSC patients, with a mean follow‐up of 25.8 months (Shinojima et al., 2020). The higher rate of SRF development in their study could be explained by the longer follow‐up. Our study has shown that hyperautofluorescence on FAF and the presence of RPE alterations and interruption of the EZ on OCT of the fellow eye at baseline of cCSC patients might be risk factors for the development of SRF at follow‐up in eyes with no SRF at baseline and no recorded previous presence of SRF. This may be helpful in counselling patients about the risks of development of bilateral disease, as patients with bilateral disease might be more prone to develop severe visual impairment (Mohabati et al., 2018; Mrejen et al., 2019). Patients with the aforementioned risk factors on OCT and FAF may therefore need more regular follow‐up as compared to patients who do not show these characteristics on OCT and FAF.

In recent years, a new clinical entity termed pachychoroid pigment epitheliopathy (PPE) has been described, which has several features similar to CSC: increased choroidal thickness, RPE alterations and/or small RPE detachments directly overlying localized areas of thickened choroid and/or dilated choroidal vessels, but without the presence of SRF (Cheung et al., 2025; Warrow et al., 2013). A study by Ersoz et al. suggested a prevalence of PPE of 61% in the fellow eyes of 282 CSC patients with unilateral SRF (Ersoz et al., 2018). Another study showed that 8/46 (17%) of eyes with a diagnosis of PPE developed CSC during a mean follow‐up period of 5 years (Karacorlu et al., 2018). It has also been suggested that degeneration of photoreceptors and/or RPE may occur even in the absence of SRF (Cheung et al., 2025; Ozdemir et al., 2018; Warrow et al., 2013). The RPE alterations observed in our study (observed in 65% of fellow eyes on OCT) might be due to PPE and could therefore have indeed increased the risk of developing SRF over time. Another hypothesis is that the RPE irregularities may have been secondary to a prior episode of SRF, which resolved spontaneously and may have remained unnoticed by the patient (Bujarborua et al., 2005).

There were no significant differences in baseline characteristics between patients who did and did not develop SRF during the course of this study, which is in line with a study by Shinojima and co‐workers (Shinojima et al., 2020). However, in the current study, corticosteroid use within 3 months prior to the onset of symptoms in the affected eye was more often present in patients who developed SRF in the fellow eye compared to patients who did not, although this finding was not statistically significant after correction for multiple testing. The most important external risk factor for developing CSC is corticosteroid use (Carvalho‐Recchia et al., 2002; Haimovici et al., 2004; Rim et al., 2018; Tsai et al., 2014). As this external risk factor has systemic effects, it could possibly increase the odds of SRF development in the fellow eye if studied in a larger population.

This study has a few limitations. First, data were collected retrospectively, which resulted in variability of follow‐up duration, which should be taken into account when interpreting the results. Due to the relatively low number of patients who developed SRF during follow‐up, we could not obtain a Kaplan–Meier survival analysis. In addition, probably due to the relatively low number of patients, some variables did show a *p*‐value lower than 0.05 but were not significant after correction for multiple testing. Moreover, patients who have had possible symptoms of CSC or claimed to have had CSC in the past were classified as ‘no history of CSC’, because these episodes were often not verified with multimodal imaging or clinical reports, despite our best efforts. Lastly, patients who do not have any visual complaints after the first presentation or recurrence of SRF are less likely to have additional follow‐up, which might be a bias that causes overestimation of the risk of development of SRF in the fellow eye. To further aid counselling in patients with unilateral cCSC, future studies are needed with a longer follow‐up period, a prospective study design, and a larger population to enable a survival analysis on SRF development in the fellow eye. Additionally, artificial intelligence strategies may improve the detection of additional risk factors for developing SRF in fellow eyes.

In conclusion, this study has shown that 7% of fellow eyes developed SRF after a mean time of 17.1 months. No statistically significant risk factors were identified after correction for multiple testing. However, certain trends were observed, including hyperautofluorescence on FAF and the presence of RPE alterations and interruption of the EZ on OCT of the fellow eye at baseline seem to occur more often in cCSC patients who develop SRF in the fellow eye. Identifying risk factors may help in better prognostication and follow‐up strategies in cCSC patients.
